# Supplementary material for: Patterns of Intron Gain and Loss in Fungi
Source: PLoS Biol. 2004 Nov 30;2(12):e422. doi: 10.1371/journal.pbio.0020422 (PMC532390; doi:10.1371/journal.pbio.0020422)
Supplement: Table S1 — Also available at http://genes.mit.edu/NielsenEtAl/. (4.3 MB ZIP). [file pbio.0020422.st001.zip › NielsenEtAl/html/306.html]

AN3390.1.NCU10045.1.MG00618.1.FG03406.1


```
 CLUSTAL W (1.82) Multiple Sequence Alignments - Introns Inserted


Sequence 1: MG00618.1	325 aa
Sequence 2: FG03406.1	326 aa
Sequence 3: NCU10045.1	347 aa
Sequence 4: AN3390.1	325 aa
Alignment Length: 355 aa
Number Identitical Residues: 96 aa
Alignment Score (without introns) 5977


MG00618.1 	---MVSLTLAILSLAAGALGAS--------RTSAPS-GCITVKSG---ASIQQAVNSLST
NCU10045.1	MKSLLLPLLSLLSLSSSALAAT--------RISPPSTACLRVGAGQPYSNIQSAVNALST
FG03406.1 	-----MKIFATLNFVTAVLAAS--------RTTAPS-GCITVKKGGQFGTIQSAVNSLST
AN3390.1  	--MRVQSYLSLFSLVGAALCAPREHFKRTARTSAPA-GCLTVGGSGTYSTIGAAFAALGS
          	        :: :.:  ..* *. .  . ::* :.*: .*: *  .   ..*  *. :*.:

MG00618.1 	TASGTQCIFLERGTYREQVLVPARKAQLTIYGYSTDTLSHNANGALITQRKSQKDGLSND
NCU10045.1	TASGSQCIFIYPGTYWEQVLIPARSAQLSIYGYTANSQGYASNTVTIIANKSQADGLSND
FG03406.1 	TASGSQCIFIDQGTYNEQVLVSARNAQLTIYGYTTDTSGYAGNKVTITAKKSQADGLNND
AN3390.1  	SSS-EACIYISAGTYKEQLTFQY-AGPLTLYGETTDTSSYKKNTVTITHTISSPEAGSLV
          	::*   **::  *** **: .    . *::** :::: .:  * . *    *. :. .  

MG00618.1 	ETATLRVKAANFRLYNVNVDNAYGEGSQAVA~LSAYAD-SGYY~GCRFTGFQDTVLANQG
NCU10045.1	QTATVRVKSSNTRLYNLNIENAHGQGSQAVA~LSVAANGVGVY~GCQLKGFQDTLLAEDG
FG03406.1 	GTATLRVKAANFKLYNVNVANTYGQGSQAVA~LSAYAD-SGYY2GVALTGFQDTLLAQEG
AN3390.1  	ASATVNAAMDNFTMYNINVVNGYGKGAQAVA2LAASGERQGYY~GCQFLGYQDTLYARVG
          	 :**:..   *  :**:*: * :*:*:**** *:. .:  * * *  : *:***: *. *

MG00618.1 	KQLYARCLVQG~ATDFVFGQYAAAWFEKNDVRVLGKSLGYITA1NGRLDNSNPSYYVFNN
NCU10045.1	AQLYARTLIQG~ATDFIFGQRAPAWFEKCDIKVMAANLGYITA1LGRDSSSNPANYVFNS
FG03406.1 	YQLYSKCMIAG~ATDFIFGQRASAWFEKNDLRVVSASVGYITA1NGRDSDSNPSNYVFNN
AN3390.1  	VQYYSNCYIEG1AVDYIFGD-ASAWFGECDI--VSNGAGYITA~MSRETASDPAWYCFDH
          	 * *:.  : * *.*::**: *.*** : *:  :. . *****  .*   *:*: * *: 

MG00618.1 	CNVAAASGQSVPRGAYYLGRPWREFARVVFQNTAMTEVINSAGWKTWNTGDERISNVLFG
NCU10045.1	CSIAAAAGNNVPSGAYYLGRPWRQFARVTFQKTSMTNVINPAGWSIWQNGDARTSNVEFG
FG03406.1 	CNIAAAAGNNVANGAYYLGRPWREYSRVTFQKTSMSAVIHPAGWSIWNKGEENTGHVQFS
AN3390.1  	CNIYGKSGLDLT-GDVYLGRPWRVLARVIYQNSELSDIINAAGWTTMAEG----ATPLYY
          	*.: . :* .:. *  *******  :** :*:: :: :*:.***.    *    .   : 

MG00618.1 	EYANSGAGSQGQRASFSKKLGSAVGIDSILGSGHRSAAFYDGSYM~--------------
NCU10045.1	EYANTGAGASGVRAWFGKLLGSPVKIESVLGSRYSRQGYYDASYI2QLSNRTATTDQDLK
FG03406.1 	EFGNTGAGSTGARASFSKKLSSAVSISTVLTSSYASKGFYDASYM~--------------
AN3390.1  	EIGNTGDGADTSKRLYLSEISAAVTKATVLGSDWT--DWLDWSY-~--------------
          	* .*:* *:   :  : . :.:.*   ::* *      : * **                

MG00618.1 	
FG03406.1 	
NCU10045.1	
AN3390.1  	
          	
```
